# Podocyte Pathology and Nephropathy – Sphingolipids in Glomerular Diseases

**DOI:** 10.3389/fendo.2014.00127

**Published:** 2014-07-30

**Authors:** Sandra Merscher, Alessia Fornoni

**Affiliations:** ^1^Peggy and Harold Katz Family Drug Discovery Center and Division of Nephrology, Department of Medicine, University of Miami, Miami, FL, USA

**Keywords:** sphingolipid, podocyte, kidney disease, glomerular disease, S1P, ASMase, SMPDL3b, ceramide

## Abstract

Sphingolipids are components of the lipid rafts in plasma membranes, which are important for proper function of podocytes, a key element of the glomerular filtration barrier. Research revealed an essential role of sphingolipids and sphingolipid metabolites in glomerular disorders of genetic and non-genetic origin. The discovery that glucocerebrosides accumulate in Gaucher disease in glomerular cells and are associated with clinical proteinuria initiated intensive research into the function of other sphingolipids in glomerular disorders. The accumulation of sphingolipids in other genetic diseases including Tay–Sachs, Sandhoff, Fabry, hereditary inclusion body myopathy 2, Niemann–Pick, and nephrotic syndrome of the Finnish type and its implications with respect to glomerular pathology will be discussed. Similarly, sphingolipid accumulation occurs in glomerular diseases of non-genetic origin including diabetic kidney disease (DKD), HIV-associated nephropathy, focal segmental glomerulosclerosis (FSGS), and lupus nephritis. Sphingomyelin metabolites, such as ceramide, sphingosine, and sphingosine-1-phosphate have also gained tremendous interest. We recently described that sphingomyelin phosphodiesterase acid-like 3b (SMPDL3b) is expressed in podocytes where it modulates acid sphingomyelinase activity and acts as a master modulator of danger signaling. Decreased SMPDL3b expression in post-reperfusion kidney biopsies from transplant recipients with idiopathic FSGS correlates with the recurrence of proteinuria in patients and in experimental models of xenotransplantation. Increased SMPDL3b expression is associated with DKD. The consequences of differential SMPDL3b expression in podocytes in these diseases with respect to their pathogenesis will be discussed. Finally, the role of sphingolipids in the formation of lipid rafts in podocytes and their contribution to the maintenance of a functional slit diaphragm in the glomerulus will be discussed.

Sphingolipids, more precisely sphingomyelin, cerebroside, and cerebrosulfatide were first described in 1884 by Johann L. W. Thudichum who named them for their enigmatic (“Sphinx-like”) properties (1). They are important components of the lipid rafts in plasma membranes of mammalian cells and thus contribute to the proper function of cells. Within the kidney, the function and survival of major cell constituents of the glomerular filtration barrier, i.e., podocytes, heavily depends on the integrity of lipid rafts. Podocytes are differentiated cells of the kidney glomerulus consisting of a cell body, major processes, and foot processes (FP). The FP of podocytes are linked to the glomerular basement membrane with their actin cytoskeleton. Processes from neighboring podocytes form a characteristic interdigitating pattern that leaves filtration slits in between them. The latter are bridged by the slit diaphragm (SD) that together with the glomerular basement membrane and the fenestrated endothelium plays an important role in the selective permeability of the filtration barrier of the glomerulus (2–4). Integrity of this filtration barrier is important in order to prevent the loss of protein into the urine (proteinuria) and mutations in genes coding for SD proteins cause proteinuria-associated nephropathies (5–9). Research of the past two decades has revealed an essential role of sphingolipids in glomerular disorders with podocyte involvement.

This review will focus on different types of sphingolipids and sphingolipid metabolites that have been implicated in the pathogenesis of sphingolipidoses of genetic and non-genetic origin with podocyte involvement. We will also discuss sphingolipid signaling in podocytes and its influence on the actin cytoskeleton.

## Biology of Sphingolipids

**Figure 1:**
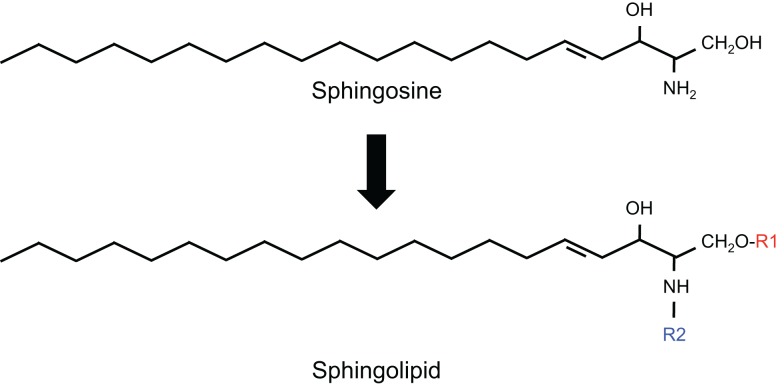
**Structure of sphingolipids**. In sphingolipids, the hydrophobic region consists of a longchain sphingoid base with generally 18 carbons, such as sphingosine, which is linked to the acyl group of a fatty acid via an amide bond (R2). The hydrophilic region (R1) consists in the simplest case of a hydroxyl group in the case of ceramide.

Sphingolipids are a diverse class of lipids with a varying degree of hydrophobic and hydrophilic properties. The hydrophobic region of sphingolipids consists of a longchain sphingoid base with generally 18 carbons, such as sphingosine, which is linked to a fatty acid via an amide bond. The hydrophilic region consists in the simplest case of a hydroxyl group in the case of ceramide. Fatty acids may vary in their composition but palmitic (C16:0) and stearic (C18:0) are most commonly present. More complex sphingolipids have sugar residues (glycosphingolipids) and phosphates as side chains (phosphosphingolipids) (Figure 1).

Glycosphingolipids such as GM1 and phosphosphingolipids such as sphingomyelins are commonly found in eukaryotes, in some prokaryotes, and in viruses as components of plasma membranes and membranes of organelles such as lysosomes, endosomes, endoplasmatic reticulum (ER), and others. The fluidity of the plasma membrane is tightly regulated by ordered packing of cholesterol between the phospholipid molecules, mainly sphingomyelin (SM) and thus sphingolipids have an important structural function. Sphingolipids are localized in the outer leaflet of the plasma membrane where they are asymmetrically distributed. Lipid rafts or raft-related caveolae are sphingomyelin-rich microdomains of the membrane, which are also enriched with cholesterol and membrane-associated proteins. The formation of lipid rafts is critical for proper cell function, protein–protein interactions, and signal transduction. For example, conversion of SM to ceramide, locally at the plasma membrane, by sphingomyelinases (SMases) will have a direct effect on the biophysical properties of the membrane and cell function as ceramide accumulation will lead to the displacement of cholesterol from the plasma membrane thus altering lipid rafts and signaling properties (10–12). Likewise, an interruption of raft-dependent cell signaling or even cell death can occur as a consequence of a cellular depletion of cholesterol that can be achieved by the use of cholesterol-depleting agents such as beta-cyclodextrin, methyl-beta-cyclodextrin, or 2-hydroxy-propyl-beta-cyclodextrin.

In recent years, it has become clear that, besides being integral part of membranes and having a structural function, sphingolipid metabolites such as ceramides, sphingosine, sphingosine-1-phosphate (S1P), and others play also important roles as second messengers in many biological processes including cell growth (13), differentiation, migration, and apoptosis (14). Complex sphingolipids were shown to interact with growth factor receptors, extracellular matrix, and neighboring cells (15). In addition, studies in yeast mutants revealed that sphingolipids have an important role in cellular stress responses as sphingolipid mutants yeast grew normally under usual culture conditions but were unable to survive if challenged or stressed (16).

**Figure 2:**
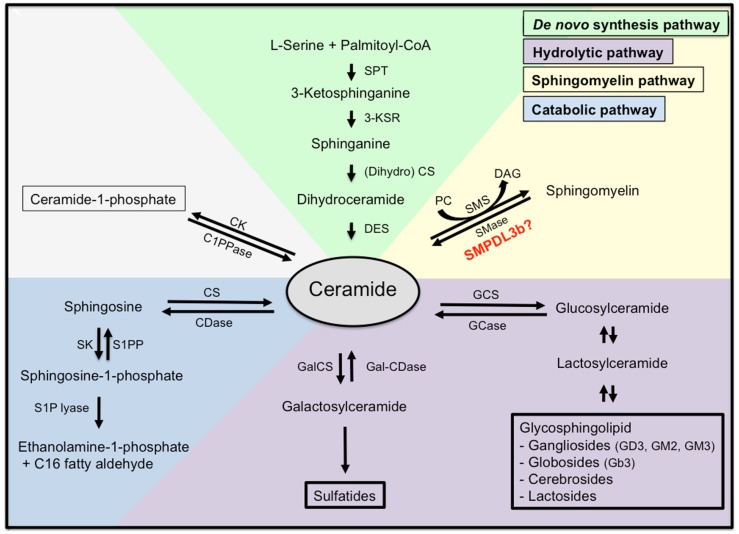
**Sphingolipid metabolism**. Ceramide is the centerpiece of the sphingolipid metabolic pathway and can be synthesized *de novo* from l-serine and palmitoyl-CoA (green), through hydrolysis of sphingomyelin (yellow), or through hydrolysis of glycosphingolipids and sulfatites (purple). Ceramide can also be synthesized from sphingomyelin through the action of sphingomyelinases, or from ceramide-1-phosphate through the action of ceramide-1-phosphate phosphatase. Finally, ceramide can be further catabolized (blue) to sphingosine and sphingosine-1-phosphate, which are biologically active metabolites and finally to ethanolamine-1-phosphate and C16 fatty aldehydes. SPT, serine palmitoyl transferase; 3-KSR, 3-ketosphinganine reductase; CS, ceramide synthetase; DES, dihydroceramide desaturase; SMase, sphingomyelinase; SMS, sphingomyelin synthetase; PC, phosphatidylcholine; DAG, diacylglycerol; C1PPase, ceramide-1-phosphate phosphatase; CK, ceramide kinase; CDase, ceramidase; CS, ceramide synthase; SK, spingosine kinase; S1PP, spingosine-1-phosphate phosphatase; GCS, glycosylceramide synthase; GCase, glycosylceramidase; GalCS, galactosylceramide synthase; Gal-CDase, galactosylceramidase.

Ceramide represents the centerpiece of the sphingolipid metabolic pathway reviewed in (17). Ceramide can be synthesized *de novo* starting with the condensation of l-serine and palmitoyl-CoA by serine palmitoyl transferase (SPT) to generate 3-ketodihydrosphinganine. The latter is then reduced by 3-ketosphinganine reductase to sphinganine, which in turn is N-acylated by ceramide synthetase (CS) to produce dihydroceramide. Finally, dihydroceramide is converted to ceramide by the enzyme dihydroceramide desaturase. Ceramide can also be generated by hydrolysis from sphingomyelin (SM) by SMases producing ceramide and phosphocholine. For sphingolipid biosynthesis, ceramide can be converted to sphingomyelin. This reaction is catalyzed by sphingomyelin synthetase (SMS), an enzyme that transfers the phosphocholine head group from phosphatidylcholine (PC) onto ceramide simultaneously generating diacylglycerol (DAG). Finally, ceramide can be generated by breakdown of glycosphingolipids and galactosylceramide to dihydroceramide and subsequent hydrolyzation (Figure 2).

Once ceramide is generated, it can accumulate in the cell or it may be further metabolized. Phosphorylation by ceramide kinase will generate ceramide-1-phosphate whereas deacylation by neutral or acid ceramidases will generate sphingosine, which can be phosphorylated by sphingosine kinase to yield S1P. Endproducts of the ceramide catabolic pathway are ethanolamine-1-phosphate and C16 fatty aldehydes, which are generated from S1P lyase from S1P.

## Sphingolipids in Glomerular Diseases

In the past decade, it has become clear that there is an association between the accumulation of sphingolipids in the kidney and glomerular disease. The accumulation of sphingolipids in form of glycosphingolipids, ceramide, and ceramide metabolites has been described in several models of experimental and clinical nephropathy and is characteristic of some rare genetic glycosphingolipid (GSL) storage disorders. The observation that intracellular accumulation of sphingolipids in glomerular cells such as podocytes is also observed in the absence of genetic mutations and is associated with the development and progression of kidney disease suggests the existence of “acquired” sphingolipid storage disorders.

Most mammalian GSLs are synthesized from glucosylceramide (Figure 2) and are primarily present in the outer leaflet of the plasma membrane where they have important functions in mediating cell–cell interactions and modulating activities of proteins in their proximity. They are usually not uniformly distributed within the plasma membrane but cluster in lipid rafts (18, 19). Gangliosides are sialic acid-containing glycosphingolipids in which one or more *N*-acetlyneuraminic acids (NANA) is linked to the sugar chain and are essential components of plasma membranes (20). Gangliosides with one NANA include GM1, GM2, GM3, gangliosides with two NANAs are GD1a, GD1b, GD2, GD3, and the gangliosides GT1b and GQ1 are characterized by three and four NANAs, respectively. Gangliosides were first identified in nervous tissue but are also abundantly present in the kidney (21). GM1, GM2, GM3, GD1a, GD1b, GD2, GD3, GT1a, and GT1b are gangliosides present in normal rat glomeruli (22–24). GM3, GD3, and disialosyllactosylceramide (*O*-acetyl GD3) are the most abundant gangliosides present in kidney and 9-*O*-acetyl GD3 is a podocyte specific ganglioside (25, 26).

## Sphingolipid Accumulation and Glomerular Disease of Genetic Origin

Sphingolipidoses are inherited disorders leading to defects in the sphingolipid metabolism resulting in the accumulation of excess glycosphingolipids and phosphosphingolipids. It is interesting to note that different metabolites will tend to accumulate in different cell types, therefore leading to highly variable clinical–pathological findings.

Gaucher disease type 1 (OMIM #230800) is the most prevalent GSL storage disease and is characterized by an accumulation of glucocerebroside (GlcCer) in the affected tissues and cells, mainly in red blood cells, liver, and spleen. Gaucher disease is of autosomal recessive inheritance and it is caused by mutations in the acid beta-glucosidase 1 (*GBA1*) gene on chromosome 1q22 in the vast majority of patients (Table 1). This gene encodes for the enzyme that cleaves the beta-glucosidic linkage of glycosylceramide and mutations in this gene lead to accumulation of GlcCer. However, in some patients GlcCer accumulation is due to a lack of saposin C. Enzyme replacement therapy with macrophage-targeted recombinant human glucocerebrosidase is successfully used to treat patients with Gaucher disease (27–29) and drugs that block GlcCer synthesis are currently being tested in clinical trials (30). Although the presence of renal pathology in Gaucher disease is rather rare, it has been described in some patients (31) and it is associated with the accumulation of GlcCer in form of Gaucher bodies in glomerular mesangial and endothelial cells and in interstitial cells of the kidney (31). Sphingolipid activator proteins (saposins A, B, C, and D) are glycoproteins that are encoded in tandem and are derived from a common precursor protein (prosaposin, PSAP). Saposins stimulate the degradation of GSLs by lysosomal enzymes. Defects in saposins are associated with the accumulation of lipids in affected tissues in lysosomal storage disorders. Humans with saposin C deficiency exhibit the clinical presentation of Gaucher-like disease (32). Combined deficiency of Saposin C and D in mice led to accumulation of GSLs and ceramide in brain and kidney due to decreased β-glucosidase activity (33).

**Table 1:** **Sphingolipid accumulation in glomerular diseases of genetic and non-genetic origin**.

Disease	OMIM	Mutated gene	Chromosomal location	Sphingolipid accumulating
**SPHINGOLIPID ACCUMULATION IN GLOMERULAR DISEASE OF GENETIC ORIGIN**				
Gaucher	230800	Acid beta-glucosidase 1 (GBA1)	1q22	GlcCer
Tay–Sachs	272800	Hexoseaminidase A (HEXA)	15q23	GM2
Sandhoff	268800	Hexoseaminidase B (HEXB)	5q13	GM2
Fabry	301500	Alpha-galactosidase A (GLA)	Xq22	Gb3, Lyso-Gb3
Hereditary inclusion body myopathy 2	600737	UDP-acetylglucosamine 2-epimerase/*N*-acetylmannosamine kinase (GNE)	9p13	Hyposialylation of glycoproteins such as podocalyxin?
Niemann–Pick	257220 607616 257200	*NPC1 NPC2 SMPD1*	18q11 14q24 11p15	Sphingomyelin
Nephrotic syndrome of the Finnish type	256300	NPHS1	19q13	*O*-actetyl-GD3
**SPHINGOLIPID ACCUMULATION IN GLOMERULAR DISEASE OF NON-GENETIC ORIGIN**				
Diabetic kidney disease				GlcCer, GM3, S1P, sphingosine?
Puromycin aminonucleoside (PAN)-induced nephropathy				GD3, *O*-actetyl-GD3
HIV-associated nephropathy (HIVAN)				Gb3
Focal segmental glomerulosclerosis (FSGS)				Sphingomyelin
Acute ischemia reperfusion injury				Ceramide

Tay–Sachs disease (OMIM #272800) is a genetic disorder with autosomal recessive inheritance that is caused by mutations in the alpha subunit of the hexoseaminidase A (*HEXA*) gene on chromosome 15q23. Sandhoff disease (OMIM #268800) is caused by mutations in the beta subunit of the hexosaminidase gene B (HEXB) on chromosome 5q13 (Table 1). Both disorders are characterized by an accumulation of the ganglioside GM2 in the affected tissues and are clinically indistinguishable from each other. GM2 accumulation occurs mainly in the brain and liver but has also been found in the kidney (34, 35). Unlike in humans, targeted inactivation of the *Hexa* and *Hexb* gene in mice revealed phenotypical differences between the two models. Whereas *Hexa* knockout mice showed GM2 accumulation in the brain and membranous cytoplasmatic bodies in neurons in the absence of neurological manifestations, *Hexb* knockout mice showed profound neurological disturbances. These differences, which are not found in patients with the two diseases, are due to differences in the ganglioside degradation pathway between humans and mice (36).

Fabry disease (OMIM #301500) is caused by mutations in the gene encoding alpha-galactosidase A (*GLA*) on chromosome Xq22, which leads to the systemic accumulation of globotriaoslyceramide (Gb3) (Table 1) and related glycosphingolipids in body fluids and affected tissues (37), mainly brain, heart, and kidney. Elevated levels of Gb3 are detected in plasma or urine of patients with Fabry disease (38, 39) and more recently, highly increased levels of globotriaosylsphingosine (lyso-Gb3) were also described (40, 41). In the kidney, Gb3 accumulation occurred mainly within lysosomal, ER, and nuclear markers of renal cells (42). The renal pathology observed in Fabry disease includes hypertrophic podocytes with foamy appearing vacuoles, characteristic inclusion bodies of glycolipids in podocytes (zebrabodies), and mesangial widening (43). Progressive podocyte injury due to accumulation of Gb3 and related glycosphingolipids was shown to be associated with albuminuria and foot process effacement. Thereby, podocyte Gb3 inclusion volume density and foot process effacement increased with age when compared with controls and correlated directly with proteinuria (44, 45). Enzyme replacement therapy using recombinant human α-GalA is the primary treatment for patients with Fabry disease and was shown to attenuate renal complications, halt the progression of renal pathology, and prevent renal failure in patients with Fabry disease (44, 46). Studies in the alpha *GalA* knockout mouse, a mouse model of Fabry disease, revealed reduced levels of glycosylceramide and ceramide in plasma, liver, spleen, kidney, and heart possibly a consequence of Gb3 accumulation. The observation that enzyme replacement therapy in this model normalized glycosylceramide levels possible via increased Gb3 degradation further supported the hypothesis that Gb3 accumulation contributes to the phenotype observed in these mice (46). Thereby, targeting of recombinant α-GalA requires the expression of endocytic receptors, megalin, sortilin, and mannose-6-phosphate receptor (M6PR), which are expressed in human glomerular podocytes (47). Interestingly, lentiviral knockdown of α-GalA in human podocytes led to intracellular Gb3 accumulation, which was associated with a loss of mTOR kinase activity and dysregulated autophagy suggesting a link between autophagy and glomerular injury in Fabry disease (48).

Hereditary inclusion body myopathy 2 (HIBM2) (OMIM #600737) is a genetic disorder with autosomal recessive inheritance that is caused by mutations in the gene encoding UDP-acetylglucosamine 2-epimerase/*N*-acetylmannosamine kinase (GNE) on chromosome 9p13 (Table 1). GNE is a key enzyme in the sialic acid biosynthetic pathway, which catalyzes the first two steps in NANA biosynthesis, which are main components of gangliosides (49). The disease is a progressive neuromuscular disorder but no renal disorders have been reported in patients with HIBM2. Interestingly, knockin mice carrying a homozygous M712T Gne/Mnk mutation died perinatally in the absence of myopathic features but were characterized by a renal phenotype. The renal pathology observed included glomerular hematuria, segmental splitting of the glomerular basement membrane, proteinuria, and podocytopathy, including effacement of podocyte FP, and reduced sialylation of the major podocyte sialoprotein, podocalyxin. ManNAc administration to homozygous knockin mice was associated with improved renal histology, increased sialylation of podocalyxin, and increased Gne/Mnk protein expression and Gne-epimerase activities (50). Likewise, mice with a V572L point mutation in the GNE kinase domain show no apparent myopathies or motor dysfunctions but exhibited renal impairment accompanied by massive albuminuria. Histologically, kidneys of the mutant mice showed enlarged glomeruli with mesangial matrix deposition, leading to glomerulosclerosis, and abnormal podocyte foot process morphologies. This phenotype was partially prevented by the administration of *N*-acetylneuraminic acid (Neu5Ac) to the mutant mice (51). These studies indicate that hyposylation of podocyte glycoproteins including podocalyxin may contribute to albuminuria, foot process effacement, and splitting of the glomerular basement membrane and that treatment with sialic acid may represent a new strategy to prevent or treat glomerular phenotypes associated with HIBM2.

Farber disease or Farber lipogranulomatosis (OMIM #228000) is a genetic disorder with autosomal recessive inheritance that is caused by mutations in the gene encoding acid ceramidase (*ASAH1*) on chromosome 8p22, the enzyme, which is responsible for the degradation of ceramide into sphingosine and free fatty acids (Table 1). Lipid accumulation is mainly seen in the joints, tissues, and the central nervous system, but also in liver, heart, and kidneys. Ceramide accumulation in the kidney leading to a particular phenotype of lipogranlulomatosis was described (52).

Niemann–Pick is a genetic disorder with autosomal recessive inheritance that is caused by mutations in the *NPC1* gene on chromosome 18q11 (OMIM #257220), by mutations in the *NPC2* gene on chromosome 14q24 (OMIM #607616), and by mutations in the sphingomyelin phosphodiesterase-1 (*SMPD1*) gene on chromosome 11p15 (OMIM #257200). Mutations in these genes lead to the accumulation of lipids in form of cholesterol (NPC1 and NPC2 mutations) and sphingomyelin (SMPD1 mutation) (Table 1). Acid sphingomyelinase (ASMase) deficiency in Niemann–Pick disease due to mutations in the *SMPD1* gene leads accumulation of sphingomyelin in the affected tissues including the kidney. The occurrence of lipid-laden macrophages resembling foam cells was described in the bone marrow, liver, and kidney in patients with Niemann–Pick disease and in SMPD1 knockout mice (53, 54). Enzyme replacement therapy using recombinant human ASM in *SMPD1* knockout mice led to significant improvements in the organs of the reticuloendothelial system but neurological deficits remained (55).

Nephrotic syndrome of the Finnish type (OMIM #256300) is a genetic disorder caused by homozygous or compound heterozygous mutations in the gene *NPHS1* encoding for the SD protein nephrin (Table 1). Nephrotic syndrome of the Finnish type occurs in association with deposits of disialoganglioside *O*-acetyl GD3 (56). Accumulation of galactosylceramides, mainly sulfatides, was also described in nephrotic syndrome of non-genetic, idiopathic orgin (57). However, what causes the accumulation of *O*-acetyl GD3 in nephrotic syndrome remains unclear. Saposins do not seem to play an important role as mRNA expression in diseased kidneys was found normal and no mutations in the PSAP gene were found in cDNA clones (56). It has been suggested that tumor necrosis factor alpha (TNFα) and CD95 may play an important role in the pathogenesis of nephrotic syndrome. TNFα and CD95 were found significantly increased in patients with nephrotic syndrome (58). It was shown in lymphoid and myeloid tumor cells that accumulation of GD3 induced Fas (APO-1/CD95)-mediated apoptosis in a caspase-independent manner that was the consequence of the disruption of the mitochondrial transmembrane potential. This phenotype was prevented by pharmacological inhibition of GD3 synthesis (59). In other studies, an important role of membrane-associated ASMase was suggested in Fas-mediated apoptosis as activation of ASMase leads to the generation of free ceramide, which then can be converted to GD3 (60, 61). A similar pathway has been recently described in TNFα-mediated apoptosis (62, 63). Significant 9-*O*-acetyl GD3 accumulation, together with increases in GM2 and GM4 gangliosides in glomerular cells was also observed after low level and long term lead exposure and was associated with decreased apoptosis in glomerular cells suggesting that GD3-O acetylation could represent a new strategy to attenuate apoptosis in renal glomerular cells and contribute to cell survival as observed during lead exposure (64).

## Sphingolipid Accumulation in Glomerular Disease of Non-Genetic Origin

Diabetic kidney disease (DKD) is the most common cause of end-stage renal disease and renal failure in the US and podocyte injury and the consequent loss of podocytes (podocytopenia) is an important feature of DKD in patients with type 1 and type 2 diabetes (65–69). Increased levels of sphingolipids such as glycosphingolipids (70), ceramide (71, 72), sphingosine (73), and sphinganine (72, 73) have been described in the plasma of patients with diabetes. More recently, it has become clear that the intracellular sphingolipid composition in podocytes and other cells of the kidney glomerulus may contribute to the pathogenesis and progression of the disease (Table 1).

Several studies investigated the effects of streptozotocin (STZ)-induced diabetes in rats on intracellular sphingolipid accumulation and its association with glomerular cell proliferation and glomerular hypertrophy. Accumulation of S1P was observed in rat glomeruli after 4 days of diabetes induction and was associated with an increase in neutral ceramidase and sphingosine kinase activity, the two enzymes involved in the conversion of ceramide to S1P (74). In another study, accumulation of GlcCer and GM3 occurred in the kidneys of rats 16 days after STZ-induced diabetes (75) whereas reduced GM3 and sialic acid content was detected in the glomeruli of rats 15 days after STZ-induced diabetes (76). Increased ceramide production due to increased expression of SPT, a key enzyme in the ceramide *de novo* synthesis pathway (Figure 2), was described in tubular epithelial cells and microvascular endothelial cells and was associated with increased apoptosis. Rapamycin treatment significantly reduced apoptosis and proteinuria indicating an important function of the Akt/mTOR pathway in STZ-induced DKD (77). We recently showed that the expression of sphingomyelin phosphodiesterase acid-like 3b (SMPDL3b) is increased in glomeruli from patients with DKD, in DKD sera treated human podocytes and in glomeruli of diabetic mice (db/db). Because SMPDL3b is a protein with homology to ASMase, we hypothesized that SMPDL3b may activate SM metabolic pathways leading to the accumulation of sphingolipids other than sphingomyelin. Increased SMPDL3b expression was associated with increased RhoA activity and apoptosis but prevented αVβ3 integrin activation via its interaction with soluble urokinase plasminogen activating receptor (suPAR) in human podocytes cultured in the presence of sera from patients with DKD and in *db/db* mice (78). Because ceramide, sphingosine, and S1P are known sphingolipid metabolites to accumulate in apoptotic cells, we determined the ceramide content in kidney cortexes of db/db mice and found ceramide levels to be decreased in kidney cortexes of these mice. We therefore concluded that increased SMPDL3b levels may lead to increased cellular sphingosine or S1P content in the kidneys of db/db mice as it is the case in glomerular mesangial and tubular cells in *db/db* mice (79, 80), and in adipocytes of *ob/ob* mice (81). Taken together, these studies indicate a possible link between sphingolipid accumulation in form of S1P, GlcCer, and GM3 and glomerular proliferation and hypertrophy in DKD whereas the accumulation of ceramide and other ceramide metabolites such as sphingosine may contribute to podocytopenia observed in DKD. Thus, targeting sphingolipids and sphingolipid metabolites may represent a new strategy to treat patients with DKD.

Puromycin aminonucleoside (PAN) induced nephropathy is a model for human minimal change disease. Following PAN injection in rats, significant decreases in kidney GD3 and *O*-acetyl GD3 occurred in a dose- and time-dependent manner and preceded the development of proteinuria indicating a possible causative effect (82). Because sialoglycoproteins contribute significantly to the negative charge of the glomerular filtration barrier, it seems possible that decreases of GD3 and *O*-acetyl GD3 contribute to decreases in the negative charge of the filtration barrier and to the changes in glomerular permeability observed in PAN-induced nephropathy (83). Likewise, it was shown that PAN treatment of human podocytes led to a loss of sialic acid which was accompanied by increased generation of superoxide anions, a phenotype that was prevented by sialic acid supplementation (84).

HIV-associated nephropathy (HIVAN) is the classic renal disease associated with HIV infection. HIV-1 infection of renal tubular and glomerular podocytes leads to dedifferentiation and increased proliferation of podocytes (85, 86). Because podoyctes do not express HIV-1 receptors, it has been suggested lipid raft mediated endocytosis may facilitate the viral entry (87) underlining an important role for sphingolipids in mediating viral entry into the host cell. Most of our understanding of the pathogenesis of HIVAN has come from the Tg26 transgenic mouse model in which the gag/pol-deleted HIV-1 provirus is expressed. Transgenic mice show glomerular epithelial cell dedifferentiation and proliferation that is associated with proteinuria and renal failure. Renal histology revealed focal segmental glomerulosclerosis (FSGS) and microcystic tubular dilatation, resembling human HIVAN (85, 88). Studies in human podocytes in culture and transgenic mice showed that stable expression of Nef was sufficient to induce increased proliferation and loss of contact inhibition (89–92). In addition, recent studies have shown a strong association between HIVAN and the *APOL1* gene on human chromosome 22 (93) and, although not found in glomeruli, significant accumulation of Gb3 was found in renal tubular epithelial cells of HIV transgenic mice (94) (Table 1) indicating a possible role for (sphingo-) lipid metabolism in HIVAN.

Focal segmental glomerulosclerosis is a glomerular disease that is characterized by proteinuria and progression to end-stage renal disease. FSGS is the leading cause of nephrotic syndrome and the most common cause of primary glomerular disease in adults (95). Several mutations in genes coding for proteins that are expressed in podocytes have been shown to cause FSGS. We will focus in this paragraph on non-genetic forms of FSGS, mainly FSGS recurrence after transplantation, which occurs in about one third of patients (96–98) and on primary (idiopathic) forms of FSGS. We recently reported an important role of sphingomyelin-like phosphodiesterase 3b (SMPDL3b) gene in FSGS. Studying 41 patients at high risk for recurrent FSGS, we showed that the number of SMPDL3b-positive podocytes in post-reperfusion biopsies was decreased in patients who developed recurrent FSGS. As mentioned above, SMPDL3b is a protein with homology to ASMase and we hypothesized that decreased expression of SMPDL3b may lead to decreased ASMase activity and accumulation of sphingomyelin contributing to the pathogenesis of FSGS (Table 1). Indeed, we were able to show that human podocytes treated with the sera from patients with FSGS had decreased SMPDL3b expression and decreased ASMase activity. In addition, this was associated with actin cytoskeleton remodeling and apoptosis, a phenotype that was prevented by overexpression of SMPDL3b in podocytes or by treatment with rituximab, a monoclonal antibody directed against CD20 that we have found to also bind SMPDL3b in podocytes. The percentage of cells characterized by actin cytoskeleton remodeling in form of a loss of stress fibers correlated with proteinuria suggesting an important role of sphingomyelin in the pathogenesis of FSGS (99). Because decreased SMPDL3b expression in podocytes *per se* does not cause actin cytoskeleton remodeling and apoptosis, it seems possible that accumulation of sphingomyelin renders podocytes more susceptible to apoptosis and may act as a master modulator of danger signaling in podocytes. Supporting an important role of SMPDL3b in actin cytoskeleton remodeling and apoptosis, it was recently demonstrated that administration of rituximab to baboons after xeno-kidney transplantation from pigs delayed, but did not prevent, the post-transplant occurrence of proteinuria. As in the case of our study investigating the role of SMPDL3b in FSGS, this study demonstrated that rituximab was also able to prevent pig podocyte injury and prevented decreases in SMPDL3b expression in podoytes after exposure to naive baboon sera in association with preservation of cell viability (100). Finally, sequestration of plasma membrane lipids by cyclodextrin was shown to prevent suPAR-mediated αVβ3 integrin activation in podocytes (101), a pathway that may be causative of proteinuria in FSGS. Because circulating suPAR levels are elevated in FSGS patients, associate with decreased SMPDL3b expression, and suPAR-dependent αVβ3 integrin activation in podocytes (78, 99, 101, 102), whereas cyclodextrin protects podoctyes from injury in DKD where SMPDL3b expression is increased in podocytes (78, 103), we investigated if SMPDL3b expression modulates the podocyte injury phenotype in these two kidney diseases. We demonstrated that contrary to what is observed in FSGS, increased SMPDL3b expression in DKD prevented αVβ3 integrin activation via its interaction with suPAR and led to increased RhoA activity rendering podocytes more susceptible to apoptosis (78). These observations suggest that SMPDL3b and thus sphingomyelin or sphingomyelin catabolites are important modulators of podocyte function shifting suPAR-mediated podocyte injury from a migratory to an apoptotic phenotype. Therefore, modulating sphingolipids in podocytes may represent a new strategy to prevent or treat podocyte injury in FSGS and DKD.

## Special Considerations Focusing on S1P and S1P Receptors in Renal Disease

Sphingosine-1-phosphate is generated by phosphorylation of sphingosine by sphingosine kinases (SPHK1, SPHK2) in response to various stimuli including growth factors, cytokines, G-protein-coupled receptor agonists, antigens, and others (Figure 2). Examples of factors that can transiently increase levels of S1P are TNFα and factors such as angiogenic growth factor, platelet derived growth factor (PDGF), and vascular endothelial growth factor (VEGF) all of which have been implicated in the pathogenesis of glomerular diseases. S1P signaling governs important cellular processes that determine cell fate. Thereby, extracellular S1P signaling is mediated via binding of S1P to G-protein-coupled-receptors (GPCRs). A family of five GPCRs termed S1P_1_–S1P_5_ [reviewed in Ref. (104, 105)] has been identified to date. Depending on the receptor subtype being expressed in the target cell, exogenous S1P can bind, and regulate a variety of important cellular functions including cell survival, cytoskeletal rearrangment, mitogenesis, cell differentiation, migration, and apoptosis. In the kidney, the receptors S1P_1_ (EDG1), S1P_2_ (EDG5), S1P_3_ (EDG3), and S1P_5_ (EDG8) are expressed in glomerular mesangial cells (106, 107) and whereas expression of S1P_1_, S1P_2_, S1P_3_, and S1P_4_, but not S1P_5_, was shown to be expressed in an immortalized mouse podocyte cell line (108). Increases in S1P synthesis mediated by sphingosine kinase, the use of S1P_1_ receptor agonists, such as FTY720 (an unselective S1P receptor agonist) and SEW2871 (a selective S1P_1_ receptor agonists) or by SPHK1 gene delivery were shown to protect from renal ischemia reperfusion injury (109–112), which is associated with increased ceramide expression (113–115), from DKD (108), and from various forms of glomerulonephritis (116–118) (Table 1). In addition, FTY720 and KRP-203, another S1P_1_ receptor agonist, have proven highly effective in preventing graft rejection in preclinical models of renal transplantation (119, 120). Whereas the activation of the S1P/S1P_1_ receptor pathway seems to be beneficial in the context of kidney disease, it was suggested that excessive S1P/S1P_2_ receptor pathway in renal tubular cells in DKD may play an important role in Rho kinase activation and renal fibrosis (80). Such mechanism could also explain activation of RhoA and increased apoptosis in podocytes in DKD as we previously described (78). In patients with lupus nephritis (LN), an inflammation of the kidney caused by systemic lupus erythematosus (SLE), a disease of the immune system, circulating S1P levels are increased (121). Likewise, S1P and dihydro-S1P levels in serum and kidney tissues from a mouse model of LN were elevated and treatment of these mice with a specific SPHK2 inhibitor, ABC294640 improved renal injury (122). It was suggested that in the case of renal inflammatory disease, extracellular S1P induces COX-2 expression via activation of S1P2, subsequently leading to Gi and p42/p44 MAPK-dependent signaling in renal mesangial cells. Although research of the past two decades has greatly advanced our understanding of the role of S1P and S1P/S1P receptor signaling in the pathogenesis and/or treatment of kidney diseases, more studies are needed to obtain a better and more detailed understanding of their physiological and pathophysiological significance *in vivo*. Certainly, targeting S1P/S1P receptor signaling pathways may represent a novel strategy to treat renal diseases.

## Special Considerations Focusing on the Actin Cytoskeleton in Renal Disease

The kidney glomerulus is a highly specialized structure ensuring the selective ultrafiltration of plasma so that essential proteins are retained in the blood (3). Podocytes are glomerular epithelial cells consisting of a cell body, major processes, and FP. FP from neighboring cells are bridged by a 40-μm wide extracellular structure known as the SD (123, 124). Podocyte injury is an important feature of several renal diseases, including FSGS and DKD, in which independent of the underlying disease, a reorganization of the FP structure with fusion of filtration slits and apical displacement of the SD occurs (3, 125, 126). The SD is also required to control actin dynamics, response to injury, endocytosis, and cell viability. These observations make actin the common denominator in podocyte function and dysfunction (127, 128). Regulation of the podocyte actin cytoskeleton is therefore of critical importance for sustained function of the glomerular filter (129, 130). The connection of the actin cytoskeleton to the SD is mediated by several podocyte proteins such as CD2AP, Nephrin, ZO-1, and Podocin (131–134). Lipid rafts in podocytes are critical for the dynamic functional organization of the SD. Nephrin is partially associated with podocyte lipid rafts and co-immunoprecipitates with a podocyte specific 9-O-acetylated ganglioside. Injection of an antibody against the 9-O-acetylated ganglioside causes morphological changes of the filtration slits, resembling FP effacement (135) further underlining the important of intact lipid rafts and sphingolipids in the organization of the SD. Other sphingolipids such as S1P have also been implicated in cytoskeletal remodeling. S1P was shown to induce rapid reorganization of the actin cytoskeleton resulting in stress-fiber formation in 3T3 fibroblast, which was accompanied by transient tyrosine phosphorylation of focal adhesion kinase (FAK) and of the cytoskeleton-associated protein paxillin in association with RhoA activation in 3T3 fibroblasts (136). In renal mesangial cells, the serine/threonine protein kinase LIM kinase-1 (LIMK-1) was identified, which is involved in the regulation of cytoskeletal organization, as a ceramide-induced protein (137). Shiga toxin is a bacterial toxin that induces intracellular signals in a manner that is dependent on glycolipid-enriched membrane domains, or lipid rafts. Shiga-toxin-mediated intracellular signals were shown to induce cytoskeleton remodeling in renal tubular epithelial carcinoma cells (138). VEGF and its receptors, FLK1/KDR and FLT1, are key regulators of angiogenesis. However, recently a new role for FLT1, i.e., the soluble form of FLT, sFLT has been described in podocytes where it binds to the glycosphingolipid GM3 in lipid rafts, promoting adhesion, and rapid actin reorganization (139). Taken together, these studies underline the important function of sphingolipids in the formation of lipid rafts in podocytes thus contributing to the maintenance of a functional SD under physiological conditions.

## Concluding Remarks

Sphingolipids play an important role in modulating podocyte function in glomerular disorders of genetic and non-genetic origin. Several genetic diseases are characterized by genetic mutations in genes that code for enzymes involved in the sphingolipid metabolism and are characterized by the accumulation of sphingolipids and sphingolipid metabolites in glomerular cells resulting in glomerular pathology. Thus, targeting sphingolipid metabolism in glomerular disease may prove beneficial in the treatment of proteinuric kidney diseases with glomerular involvement. Enzyme replacement therapy has proven to ameliorate disease progression in sphingolipid associated disorders of genetic origin such as Gaucher and Fabry disease. However, less is known about sphingolipid associated disorders of non-genetic origin. While ManNAc and rituximab are promising available therapeutic strategies for sphingolipid associated disorders of non-genetic origin, additional therapeutic strategies specifically targeting proteins such as SMPDL3b remain to be developed. Because sphingolipidoses of non-genetic origin seem to be more complex, additional research needs to be completed in order to elucidate the exact mechanisms by which sphingolipids cause injury to renal cells and thus contribute to the pathology of glomerular diseases.

## Conflict of Interest Statement

Sandra Merscher and Alessia Fornoni are inventors on pending or issued patents aimed to diagnose or treat proteinuric renal diseases. They stand to gain royalties from their future commercialization. Alessia Fornoni is consultant for Hoffman-La Roche, Alexion, and Mesoblast on subject matters that are unrelated to this publication.
